# An Assessment of Five (PANSS, SAPS, SANS, NSA-16, CGI-SCH) commonly used Symptoms Rating Scales in Schizophrenia and Comparison to Newer Scales (CAINS, BNSS)

**DOI:** 10.4172/2155-6105.1000324

**Published:** 2017-05-11

**Authors:** Suneeta Kumari, Mansoor Malik, Christina Florival, Partam Manalai, Snezana Sonje

**Affiliations:** Department of Psychiatry and Behavioral Sciences, Howard University Hospital, Washington DC, USA

**Keywords:** Schizophrenia, Psychopathology, Mental health, Clinical practice

## Abstract

Scales measuring positive and negative symptoms in schizophrenia remain the primary mo Scales measuring positive and negative symptoms in schizophrenia remain the primary mode of assessing and diagnosing schizophrenia by clinicians and researchers. The scales are mainly used to monitor the severity of positive and negative symptoms and track treatment response in schizophrenics. Although these scales are widely used, quality as well as general utility of each scale varies. The quality is determined by the validity and reliability of the scales. The utility of the scale is determined by the time of administration and the settings for which the scales can be administered in research or clinical settings. There are relatively fewer articles on the utility of newer scales like CAINS (Clinical Assessment Interview for Negative Symptoms) and the BNSS (Brief Negative Symptom Scale) that compare them to the older scales PANSS (Positive and Negative Symptoms Scale), SAPS (Scale for the Assessment of Positive Symptoms) SANS (the Scale for the Assessment of Negative Symptoms), NSA-16 (Negative Symptom Assessment-16) and CGI-SCH (Clinical Global Impression Schizophrenia.

The older scales were developed more than 30 years ago. Since then, our understanding of negative symptoms has evolved and currently there are newer rating scales evaluating the validity of negative symptoms. The older scales do not incorporate the latest research on negative symptoms. CAINS and BNSS are attractive for both their reliability and their concise accessible format, however, a scale that is simpler, accessible, user-friendly, that incorporates a multidimensional model of schizophrenia, addresses the psychosocial and cognitive component has yet to be developed.

## Introduction

Since Eugen Bleuler coined the term “schizophrenia” in 1908 as a name for what was originally known as “dementia praecox,” schizophrenia continues to be a disorder that remains challenging to define. As can be expected, various scales and instruments have been proposed and developed for both clinicians and researchers to screen for schizophrenia, and these different instruments reflect the different understandings of how schizophrenia can be best defined and classified in terms of its symptoms. Up until the 1980s, most researchers focused on symptoms that could be described as “positive” symptoms, such as hallucinations, delusions, and thought disorders, while generally ignoring apathy, alogia, avolition and other so-called “negative” symptoms. In 1980, however, TJ Crow's groundbreaking model of schizophrenia as a disease comprised of “two syndromes” introduced the concept of a dichotomous set of positive and negative symptoms and changed much of how researchers would later understand and screen for schizophrenia [1].

Since then, scales developed to screen for schizophrenia have primarily focused on assessing patients through the use of positive and negative symptoms. The PANSS, SAPS, and SANS are well-established scales that have been used to objectively assess for schizophrenia symptoms. The fact that it is sensitive to change makes it a “gold standard” in treatment studies. When used longitudinally, psycho-pharmacological research supports the PANSS' construct, its discriminative, convergent, and predictive validity, as well as its sensitivity. The PANSS is not designed to rate negative symptoms exclusively; rather, it is a comprehensive scale for the assessment of psychopathology. Furthermore, initially, the progress in the development of new pharmacological treatment for the negative symptoms of schizophrenia is restricted by limitations of available assessment tools. The multi-site Collaboration to Advance Negative Symptoms Assessment was established to develop and validate a new clinical rating scale, CAINS (The Clinical Assessment Interview for Negative Symptoms), to address limitations of existing measures. To the author's knowledge, there has not yet been any review article evaluating older scales (PANSS, SAPS, SANS) and comparing them with the newer scales (CAINS and BNSS).

## Objective

The main objective of this paper is to review and assess utility of well-established scales: the Scale for the Assessment of Positive Symptoms (SAPS), the Scale for the Assessment of Negative Symptoms (SANS), the Positive and Negative Symptoms Scale (PANSS), the Negative Symptom Assessment (NSA-16) and the Clinical Global Impression Schizophrenia (CGI-SCH) and to compare these scales to the newer screening tools: The Clinical Assessment Interview for Negative Symptoms (CAINS) and The Brief Negative Symptom Scale (BNSS).

## Methods

A literature review from 1980-2016 was performed using the following search engines: PubMed, First Search, Cochran, Google scholar online, EBSCO host, and psychiatryonline.org. Boolean search terms included “positive symptoms scale in schizophrenia”, “negative symptoms scale in schizophrenia”, “positive and negative syndrome scale in schizophrenia”, “screening for schizophrenia”, and “utility of scales in schizophrenia”. Research articles that were generated using the above mentioned search terms met our inclusion criteria if at least one of the negative or positive symptoms scales (PANSS, SANS, SAPS, NAS-16 and CGI-SCH CAINS, BNSS) were mentioned within the title and/or abstract. We excluded editorials.

### The Scale for the Assessment of Negative Symptoms and the Scale for the Assessment of Positive Symptoms (SANS and SAPS)

The (SANS) and (SAPS) were developed in 1980 to fill a conspicuous gap in tools that could effectively measure the severity of negative and positive symptoms [2]. A standardized scale measuring either positive or negative symptoms did not exist at the time, and negative symptoms were often overlooked, in both clinical as well as in research settings, while positive symptoms were sometimes overemphasized. With Crow's work on the importance of negative symptoms, new interest in screening patients with negative symptoms, as well as the inter-correlation of negative symptoms, arose [3]. Partly in response to this paradigm shift, the Scale for the Assessment of Negative Symptoms (SANS) was developed [4]. SAPS were subsequently released a year later, enabling the clinician to evaluate positive symptoms using a similar structure and format to SANS [5]. Specific symptoms in both scales were chosen on the basis of both clinical experience and empirical statistical evaluation of data interrelationships and correlations [6].

SANS and SAPS are both utilized frequently in clinical and research settings. The question of reliability and validity has been raised since its inception, and various studies have been conducted on the validity of the scales. Earlier studies have mostly focused on interrater reliability, which has been shown to be consistent, even in multiple cross-cultural settings [2]. Other studies have focused on the temporal stability of the two scales, particularly in regards to the effect of treatment [7]. One study conducted by Malia et al. demonstrated that while SAPS and SANS both show moderate temporal stability over a 12-month time frame, subscale scores of apathy and bizarre behavior were not shown to have much stability [8].

### Nature of scoring

SANS measures negative symptoms on a 25 item, 6-point scale. Items are listed under the five domains of affective blunting, alogia, avolition/apathy, anhedonia/asociality, and attention. While, SAPS measures positive symptoms on a 34 item, 6-point scale. Items are listed under hallucinations, delusions, bizarre behavior, and positive formal thought disorder. Items on both scales are clearly defined.

### Criticisms

While SAPS and SANS are commonly utilized throughout research to assess symptoms of schizophrenia, one pertinent criticism of these two scales strikes at the positive/negative symptoms model of schizophrenia that has been popular since the 1980s- some authors have suggested that the bi-dimensional relationship between SAPS and SANS may be confounding the ability of those who use the scales to move beyond a dualistic model of negative and positive symptoms, which in itself may be a construct that is not necessarily helpful. Advocating for a re-conceptualization of the structure of schizophrenia, Klimidis, et al. and Minas, et al. proposed a multidimensional structure composed of at least three categories, including hallucinations/delusions, positive thought disorder, and negative symptoms, rather than merely dividing schizophrenic symptoms into positive and negative symptoms [9,10]. A separate study conducted on the inter-correlations between symptoms utilizing SAPS and SANS produced a three dimensional model composed of psychotic, disorganized, and negative factors [11]. Proponents of a more complex paradigm of schizophrenic symptomatology argue that schizophrenia cannot be separated or divided as neatly as SAPS and SANS. Based on Crow's “two syndromes,” newer models that take more dimensions and incorporate the diverse elements of schizophrenic symptoms into their structures may need to be developed.

### The positive and negative symptom scale (PANSS)

PANSS provides objective measuring of clinical response to pharmacologic treatments and it is incredibly useful in clinical research, with some claiming it as the “gold standard measure of treatment efficacy.” Longitudinal data for individual patients can be pooled together to examine the effect covariates have on the treatment arm versus the control placebo group in therapy specific studies, hence, PANSS is a reliable means of assessing patients chronologically throughout the course of their illness. A study categorized patients into four mutually exclusive groups based upon results from the PANSS. These results showed that in a treatment group primarily seen in the outpatient setting, “19% of individuals were classified as having prominent negative symptoms, 20% as having prominent positive symptoms, and 21% as having both prominent positive and prominent negative symptoms” [12]. This study reinforced that those with negative symptoms have poorer overall outcomes as measured by remission rates and that those with both positive and negative symptoms have even worse outcomes, further demonstrating that the negative symptoms directly affect severity and chronicity of schizophrenia.

### Nature of scoring

PANSS is comprised of 30 distinct items organized into three independent subscales with scoring that ranges from 30 to 210 points [13]. It has been previously demonstrated that the positive, negative, and general psychopathology sub-scales show normal distribution and independence from each other. The negative symptoms subscale assesses for blunted affect, emotional withdrawal, poor rapport, passive/apathetic social withdrawal, difficulty in abstract thinking, lack of spontaneity and flow of conversation, and stereotyped thinking. The positive subscale addresses delusions, conceptual disorganization, hallucinatory behavior, excitement, grandiosity, suspiciousness, and hostility. The general psychopathology subscale addresses somatic concern, anxiety, feelings of guilt, tension, mannerisms and posturing, depression, motor retardation, uncooperativeness, unusual thought content, disorientation, poor attention, lack of judgment and insight, disturbance of volition, poor impulse control, preoccupation, and active social avoidance.

### Criticisms

In the midst of a body of literature with supportive data on the validity and usefulness of PANSS, some still question the scale's ability to serve as a “stand-alone” screen for schizophrenia, challenging its reputation for being the gold standard scale. There is a degree of ambiguity and redundancy for evaluation of cognitive items assessed through its sub-scales. The biggest pitfall of PANSS is its lack of sensitivity and specificity in predicting global cognitive functioning. Additionally, the depression sub-scale fails to differentiate between “depression, negative symptoms, and extra-pyramidal side effects” which is a crucial problem given the distinct treatments and adverse downstream sequelae if inappropriately diagnosed [13]. Evaluating the factors measured by PANSS individually in a comprehensive fashion often leads to creating lengthier scales with redundant inquiries. Conversely, however, paring down the scale to minimal inquiries is just as problematic and can result in yielding incomplete data, weaker correlations, and less reliable outcomes [13]. Also, studies that use PANSS to evaluate the efficacy of psychotropic pharmacotherapy can be biased when mean outcomes are reported, serving as a systematic flow that is unlikely to detect covariates affecting placebo response [13].

Indeed, one of the most common drawbacks of PANSS is its complexity. In addition to its length, PANSS, which utilizes an interval scale of 1 to 7 for each of its 30 items, requires converting PANSS into a ratio scale in order to score patients and track response to treatment correctly. A recent systematic review found that as many as 62% of authors utilizing PANSS may have used incorrect calculations in their research, and that very few of the articles even included calculation methods [14].

PANSS was compared with Brief Psychiatric Rating Scales (BPRS)/ older counterpart and it has shown consistently better outcome than (BPRS). In a psychiatric rehabilitation study both tools exhibited strong interrater reliability; however, result showed that PANSS was superior to the BPRS in clinical predictive power [14].

### Negative symptoms assessment 16 (NSA-16)

The original NSA-16 scale was developed by Alphs et al. in 1989 [15]. The newer truncated version, the Negative Symptoms Assessment-4 (NSA 4), was adapted from the prototype in 1993 as a validated tool for evaluating negative symptoms of schizophrenia [16]. The NSA-16 examines for the presence, severity, and range of negative symptoms associated with schizophrenia. It was meant to be a concise and easy-to-use instrument with strong psychometric properties in terms of validity, reliability, sensitivity to change, and good clinical utility.

The NSA-16 is a semi-structured interview containing 16 items that comprehensively assess the negative syndrome of schizophrenia and it includes the following factors: communication, emotion/affect, social involvement, motivation, and retardation [15]. These factors are assessed through a structured interview and are extensive and well-defined to help standardize assessment [16].

Axelrod BN, et al, [16] assessed the validity of this scale in a sample of 223 un-medicated schizophrenic inpatients. In this study, a five factor model was found to best characterize the structure of this rating scale. The study provided support for a multidimensional model of negative symptoms in schizophrenia and it offered a useful measure of negative symptoms assessment. Standardized measurement of negative symptoms was also achieved in international trials, further supporting the validity of NSA-16. Dawn Velligan et al examined whether changes in negative symptoms (NSA 16) were associated with changes in functional outcome. Results showed that the relationship between negative symptoms changes and changes in functional outcome is complex and that negative symptoms drove the changes in the social and occupation functional scale (SOFAS) rather than the reverse [15,17]

### Nature of scoring

It is a semi-structured 16 item interview, utilizing the five factors: 1. Communication, 2. Emotion/Affect, 3. Social Involvement, 4. Motivation, 5. Retardation (18). Items are rated using a 6-point Likert scale where higher scores reflect greater impairment. Detailed anchoring criteria for the rating points are provided in the scale, along with a total score, sum of the scores on the 16 item scale, and a global negative symptom rating based on the global clinical impression of the patient's negative symptoms [19].

### Criticism

The main limitation of the NSA-16 is its high reliance on functioning or behaviors, even for experiential symptoms, such as reduced social drive, whose severity is measured by type and frequency of social interactions [19]. The SANS and the NSA-16 both provide a focused assessment of negative symptoms, but they must be used in conjunction with a positive symptom rating scale [18]

### Negative symptoms assessment 4 (NSA-4)

A study published in the Int. Journal of Psychiatry about the validation of a 4-item Negative Symptom Assessment (SA-4) [20]. This study revealed NSA-4 is a short practical clinical tool for the assessment of negative symptoms in schizophrenia. The psychometric properties and predictive power of a four-item version (NSA-4) were compared with the NSA-16 to determine predictive validity and construct validity. Both scales showed acceptable internal consistency (cronbach alpha 0.85 and 0.64 respectively) and test retest reliability (intra-class correlation coefficient 0.87 and 0.82). This study demonstrates that NSA-4 ofters accuracy comparable to the NSA-16 in rating negative symptoms in patients with schizophrenia [20].

### CGI-SCH scale (The Clinical Global Impression-Schizophrenia Scale)

The CGI-SCH scale assesses the positive, negative, depressive, cognitive symptoms, and overall severity of schizophrenia [21]. The (CGI-SCH) scale, is a brief assessment instrument which is originally adapted from the Clinical Global Impression (CGI) scale and the CGI-Bipolar Patients (CGI-BP) scale [22,23]. It was developed to study the outcome of antipsychotic treatment in schizophrenia in an observational study (Schizophrenia Outpatient Health Outcomes (SOHO) Study [24]

The CGI-SCH has shown strong validity and it has slightly higher interrater reliability than that for the PANSS [25]. A study of 114 patients measuring the diversity of symptoms present in schizophrenia found high correlation coefficients between the CGI-SCH, Global Assessment of Function (GAF) and PANSS scores and substantial reliability in all dimensions, except depressive dimension. This study concluded the CGI-SCH scale is a valid, reliable instrument to evaluate severity and treatment response in schizophrenia. Administering the instrument is simple, concise, and quick, which makes it an appropriate scale for use in observational studies and in routine clinical practice [21].

### Nature of scoring

The CGI-SCH is a simpler scale as it consists of only two categories: severity of illness and degree of change. The severity of illness category evaluates the situation during the week previous to the assessment, while the degree of change category evaluates the change from the previous evaluation. Each category contains five different ratings (positive, negative, depressive, cognitive, and global) that are evaluated using a seven-point ordinal scale.

### Criticisms

The CGI-SCH lacks good interrater reliability, sensitivity to change, and low correlation coefficient for depression rating [21].

### The CAINS and BNSS (Clinical Assessment Interview for Negative Symptoms and Brief Negative Symptom Scale)

In 2005, the National Institute of Mental Health held a consensus development conference on negative symptoms. Two next-generation negative symptom scales resulted from this meeting: BNSS and CAINS. Both measures are becoming widely used and various research studies have demonstrated good psychometric properties for each scale. The study published in a schizophrenia bulletin provides the first direct psychometric comparison of these scales [26]. In this study, 65 outpatient patients diagnosed with schizophrenia or schizoaffective disorder completed clinical interviews, questionnaires, and neuropsychological testing. Separate raters completed the BNSS and CAINS within the same week. Results indicated that both measures had good internal consistency, convergent validity, and discriminate validity. High correspondence was observed between CAINS and BNSS blunted affect and alogia items. Moderate convergence occurred for avolition and asociality items, and low convergence was seen among anhedonia items. Findings from this study suggest that both scales have good psychometric properties [26].

The CAINS is an effective and validated tool for measuring negative symptoms in schizophrenia. Using a diverse sample of 162 outpatients with schizophrenia or schizoaffective disorder, the researchers assessed the structure, interpreter agreement, test-retest reliability, and convergent and discriminant validity of the 13-item tool. Results were promising. The scales demonstrated good internal consistency, test-retest stability, and interrater agreement. The CAINS also showed strong convergent validity, which was determined by linkages with other measures of negative symptoms. CAINS, though brief, is also comprehensive and employable across a wide range of research and clinical contexts [27].

A study published in Schizophrenia Research highlighted the fact that patients with schizophrenia, especially those who have persistent and clinically significant negative symptoms (PNS), have the poorest functional outcomes and quality of life [28]. The presence of negative symptoms represent an unmet therapeutic need for large numbers of patients with schizophrenia. There is not one psychosocial treatment model that has been established that could address the entire constellation of PNS. In this study, a total of 51 patients with PNS were randomized into one of two groups for a period of 9 months: 1) MOtiVation and Engagement (MOVE) or 2) Treatment as usual. MOVE was a home based multi-modal treatment that employed a number of cognitive and behavioral principles to address the broad range of factors contributing to PNS and their functional consequences. Patients were assessed at baseline and every three months with multiple measures of negative symptoms. The results from this study revealed repeated measure analyses of variance for mixed models, and indicated significant Group by Time effects for the Negative Symptom Assessment (NSA; p<0.02) and the Clinical Assessment Interview for Negative Symptoms (CAINS p<0.04). Group differences were not significant until nine months of treatment and were not significant for the Brief Negative Symptom Scale (BNSS) [28].

According to the 2005 NIMH–MATRICS consensus statement, CAINS and BNSS address the five currently recognized domains of negative symptoms, differentiate appetitive aspects of anhedonia from consummatory aspects, and address desire for social relationships. Thus far, both have exhibited promising psychometric properties [29].

The CAINS is an empirically developed and evaluated measure of negative symptoms. Findings from previous research studies indicate that the CAINS is brief yet comprehensive and employable across a wide range of research and clinical contexts. Negative symptoms are resistant to treatment and impede functional recovery in schizophrenia. Recognizing the clinical importance of negative symptoms, the top recommendation was the Consensus Development Conference on Negative Symptoms (convened by the National Institute of Mental Health (NIMH) and the Measurement and Treatment Research to Improve Cognition in Schizophrenia (MATRICS initiative) for stimulating novel treatment development [27].

### Nature of scoring

The CAINS and BNSS are two scales that explore psychometric domains, including negative symptoms, different aspects of anhedonia, and interest in social relationships with others. Both scales use 13 items to assess negative symptoms [27]. It is anticipated that prospective clinical trials enrolling those with negative symptoms will demonstrate the relative sensitivity to change and global suitability of the BNSS and CAINS vs. each other and the earlier generation scales [30]. Multiple studies have found that regardless of the scale used to assess negative symptoms, strong correlations exist between higher negative symptom scores and poorer social functioning [27,28,30] Overall CAINS and BNSS are attractive for both their reliability and their concise accessible format.

### Criticisms

CAINS and BNSS continue to evaluate patients' primary diagnosis on the basis of negative symptoms, with no integration of other aspects of the patients' social and cognitive functioning. The common critique leveled at SAPS and SANS for being too restrictive can also be applied to both CAINS and BNSS, and multidimensional scales has yet to be developed. Furthermore, CAINS scales are not strongly related to depression, agitation, or positive symptoms [27].

## Conclusion

The older scales were developed more than 30 years ago. Since then, our understanding of negative symptoms has been evolved and currently there are newer rating scales reviewing the validity of negative symptoms. The older scales questionnaire does not incorporate the latest research on negative symptoms established by the NIMH consensus development conference on negative symptoms (CAINS and BNSS). This is the biggest difference between the older and newer scales.

It is clear that the newer negative symptom scales represent progress in the understanding of schizophrenia psychopathology. However, they still neglect to address the psychosocial and cognitive factors that are useful outcome measures.

While there are many different scales available to assess positive and negative symptoms of schizophrenia, a scale that is simpler, accessible, user-friendly, incorporates a multidimensional model of schizophrenia, addresses the psychosocial and cognitive component, and helps us better understand the severity and psychopathology of schizophrenia has yet to be developed.

## Figures and Tables

**Table 1:** Schizophrenia rating scales.

Instrument (Author, Year)	Administration Time	Type of measure	Number of Items	Strengths	Weakness	General Utility
Positive and Negative Syndrome Scale. (PANSS; Kay et al., 1987) [31].	45-50 mins	Option of both PANSS: clinician-completed, SCI-PANSS: interview IQ-PANSS: observer-completed	Total of 30 items. (7) Constitute a Positive Scale, (7) negative scale and (16) general psychopathology scale.	The fact that it is sensitive to change makes it a “gold standard” in treatment studies. Psycho-pharmacological research supports the PANSS' construct, discriminative, convergent, and predictive validity, as well as its drug sensitivity, when used longitudinally. The PANSS is not designed to rate negative symptoms exclusively, rather, it is a comprehensive scale for the assessment of psychopathology (Kay et al., 1987) [31].	Outdated, lengthy. PANSS and SANS have been criticized (Blanchard et al 2011) because they include items that measure cognitive functioning (attention bias or abstract thinking), which have been now recognized as a distinct category from negative symptoms (Harvey et al 2006).	Most commonly used ratings scale. Widely used to assess response to antipsychotic therapy. Commonly used in both academic and pharmaceutical industry trials.
Scale for Assessment of Positive Symptoms. SAPS- (Andreasen, 1984) [32].	30 Min	Clinician rated.	Total of 34 items, measures hallucinations, delusions, bizarre behavior and thought disorder.	Recognizes positive symptoms. Has good validity and inter-rater reliability for positive symptoms (Andreasen et al., 1984) [32].	Cannot be used alone. Used in conjunction with SANS.	Screening scale for assessment of positive symptoms. Scale for rating the severity of positive symptoms (Andreasen, 1984) 33*.
The Scale for Assessment of Negative Symptoms (SANS), (Andreasen, 1983) [33].	Cannot be measured It varies.	Clinician rated.	The SANS as originally published had 25 items. Currently, SANS consists of 19 items representing 5 scales: Affective Flattening or Blunting Alogia Avoliton-Apathy Anhedonia-Asociality Inattention. (Andreasen, 1983) [34]	Separates negative symptoms from positive symptoms and depression.	Cannot be used alone; need SAPS.	Most commonly used ratings scale. SANS helps the clinician track treatment progress. It is widely used in both academic and pharmaceutical industry trials.
Clinical Assessment Interview for Negative Symptoms (CAINS). CAINS-2010 was Developed by CANSAS Group. CAINS and BNSS were developed following a National Institute of Mental Health consensus meeting and addressed some of the shortcomings of earlier instruments. (Kring 2010) [35]. This represents an important and novel addition. CANSAS: (Collaboration to Advance Negative Symptom Assessment in Schizophrenia)	Cannot be measured. It varies.	Clinician rated. It is comprised of two scales that are scored separately. Motivational and pleasure scale (Nine- items) and Expression Scale (four-items) 1) Facialexpression 2) Vocalexpression 3) Expressivegestures 4) Quality ofspeech.	Total of 13 items that assess the presence and severity of negative symptoms. It provides standardized interview probes and descriptive anchor points. All Items are scored on a five-point scale from 0 (no impairment) to 4 (severe deficit)	Items in the CAINS construct cover approach motivation, pleasure, social engagement, affective expression, behavioral engagement, and comprehensive assessment of negative symptoms. CAINS is a brief yet comprehensive scale and employable across a broad range of clinical and research contexts. It is a welldeveloped and evaluated scale for measuring negative symptoms. It demonstrates good internal consistency, test-retest stability, and interrater reliability/ agreement. It also demonstrated greater convergent validity than the BPRS and SANS for negative symptoms (Kring, 2010) [35].	CAINS scales are not strongly related to depression, agitation or positive symptoms (Kring, 2010) 35*.	CAINS represents a state of the art approach to negative symptoms. Developed for treatment trials, but can be used in other types of negative symptoms. The Clinical Assessment Interview for Negative Symptoms (CAINS) is yielding promising results in the clinical and research setting.
Brief Negative Symptom Scale (BNSS) (Kirkpatrick, 2011) [36].	15 minutes	Clinician rated. Measures negative symptoms in a multicenter clinical trial. In addition to distress, it addresses the same above five negative symptoms domain included in CAINS. (Kirkpatrick, 2011) [36].	13 items organized into 6 subscales. Blunted affect Alogia Asociality Anhedonia Distress	Its design enables researchers to consider many aspects of negative symptoms. The BNSS scores are highly correlated with SANS and PANSS negative symptoms scores. It has strong interrater, test-retest and internal consistency. (Kirkpatrick, 2011) [36].	Need to know if BNNS is sensitive to change (Its unknown if it could be used in clinical trials)	BNSS was developed as a concise instrument suitable for a multicenter clinical trial. (Kirkpatrick, 2011) 36*. Both the BNSS and CAINS represent state of the art approaches to negative symptoms and are yielding promising results in the clinical and research settings. (Kirkpatrick, 2011) 36*
Negative Symptoms Assessment-4 NSA -4 (Alphs, 2010) 40*	Rapid testing Includes 4 questions.	Clinician rated Requires brief training (Alphs, 2010) [40].	Four items from NSA-16. Restricted speech quality Reduced range of motion Reduced social drive Reduced intent	Offers accuracy comparable to the NSA-16 in rating negative symptoms in patients with schizophrenia, Good predictive validity and construct validity, Internal consistency and test--retest reliability, High correlation with other measures of negative symptoms, demonstrating convergent validity. (Alphs, 2011) [41].	Lesser correlations with measures of other forms of psychopathology. (Alphs, 2010) [40].	NSA-4 as a practical clinical tool for assessing the severity of negative symptoms in patients with schizophrenia and tracking their course over time. (Alphs, 2010) 40*
Clinical Global Impression-Schizophrenia (CGI-SCH) Adapted from the Clinical Global Impression (CGI) scale (Guy W, ed.) [42]. CGI-Bipolar Patients (CGI-BP) scale (Spearing MK) [43]. The scale was developed as a European International Project	30 Minutes	Clinician rated	Two categories: Severity of illness Degree of change, Each category contains five different ratings Positive Negative Depressive Cognitive Global evaluated using a seven-point ordinal scale.	Simple, concise, and quick to administer. Higher reliability than that of the Positive and Negative Symptoms Scale PANSS; (Kay, Fizzbein, & Opler,1987) [45] and General Assessment of Functioning GAF; (Jones et al., 1995) Strong Validity and good psychometric properties, Interrater Reliability, correlation coefficient, sensitivity similar to PANSS (Haro JM, 2003) [44]. The CGI-SCH global score assesses global severity of the disorder, including both symptoms and interference with functioning. (Haro JM, 2003) [44].	Lacks good interrater reliability, sensitivity to change, and has a low correlation coefficient in depression rating. (Haro JM, 2003) [44].	The CGI-SCH scale is a valid reliable instrument for evaluating severity and treatment response in schizophrenia. Its simplicity and quick administration time make it appropriate for use in routine clinical practice and in observational studies. (Haro JM, 2003) 44*.
